# Transcriptional Regulation by Competing Transcription Factor Modules

**DOI:** 10.1371/journal.pcbi.0020164

**Published:** 2006-12-01

**Authors:** Rutger Hermsen, Sander Tans, Pieter Rein ten Wolde

**Affiliations:** FOM Institute for Atomic and Molecular Physics, Amsterdam, The Netherlands; University of California San Francisco, United States of America

## Abstract

Gene regulatory networks lie at the heart of cellular computation. In these networks, intracellular and extracellular signals are integrated by transcription factors, which control the expression of transcription units by binding to *cis*-regulatory regions on the DNA. The designs of both eukaryotic and prokaryotic *cis*-regulatory regions are usually highly complex. They frequently consist of both repetitive and overlapping transcription factor binding sites. To unravel the design principles of these promoter architectures, we have designed in silico prokaryotic transcriptional logic gates with predefined input–output relations using an evolutionary algorithm. The resulting *cis*-regulatory designs are often composed of modules that consist of tandem arrays of binding sites to which the transcription factors bind cooperatively. Moreover, these modules often overlap with each other, leading to competition between them. Our analysis thus identifies a new signal integration motif that is based upon the interplay between intramodular cooperativity and intermodular competition. We show that this signal integration mechanism drastically enhances the capacity of *cis*-regulatory domains to integrate signals. Our results provide a possible explanation for the complexity of promoter architectures and could be used for the rational design of synthetic gene circuits.

## Synopsis

Transcription regulatory networks are the central processing units of living cells. They allow cells to integrate different intracellular and extracellular signals to recognize patterns in, for instance, the food supply of the organism. The elementary calculations are performed at the *cis*-regulatory domains of genes, where transcription factors bind to the DNA to regulate the expression level of the genes. The logic of the computations that are performed depends upon the design of the *cis*-regulatory region. Not only in eukaryotic cells, but also in prokaryotic cells, the architectures of the *cis*-regulatory regions are often highly complex. They often contain long arrays of transcription factor binding sites. Moreover, the binding sites often overlap with one another. Hermsen, Tans, and ten Wolde discuss whether such complex architectures can be explained from the basic function of *cis*-regulatory regions to integrate signals. The authors combine a physicochemical model of prokaryotic transcription regulation with an evolutionary algorithm to design *cis*-regulatory constructs with predefined elementary functions. The resulting architectures make extensive use of repeating binding sites that are organized into cooperative modules. More surprisingly, these modules often overlap with each other, leading to competition between them. This interplay between intramodular cooperativity and intermodular competition is a powerful mechanism to achieve complex functionality, which may explain the daunting complexity of promoter architectures found in nature.

## Introduction

**Figure 1:**
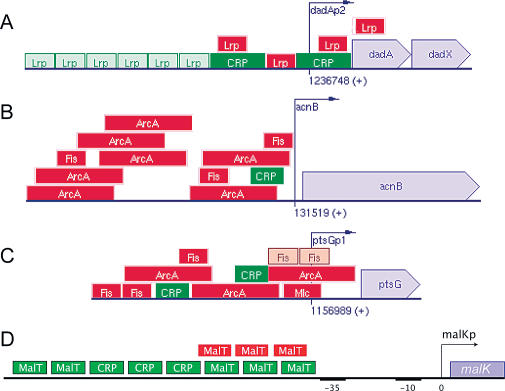
Examples of Complex E. coli Promoters (A–C) Taken directly from the EcoCyc database [8]. (D) Described in [25]. Green blocks denote TF binding sites that have an activating effect; red blocks denote repressor sites. Brown sites can both activate and repress transcription. Note that repetitive and overlapping binding sites occur frequently. Understanding these kinds of promoters requires detailed quantitative information about binding affinities and interactions.

**Figure 2:**
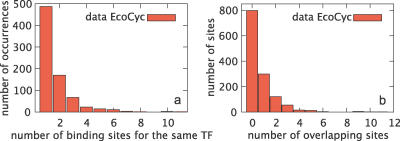
Repetitive and Overlapping Binding Sites (A) Histogram of the number of binding sites responsible for each interaction between a TF and an operon, according to the EcoCyc database [8]. Note that multiple sites are common; the *cis*-regulatory region of *focA,* e.g., has as many as 11 binding sites for NarL. (B) Histogram of the number of binding sites overlapping with each binding site [8]. For example, bin 1 with height 300 should be interpreted as: there are 300 binding sites that overlap with exactly one other binding site. Overlap is common; some ArcA sites in the *sodA* regulatory region overlap with as many as 11 sites.

Cells continually have to make logical decisions. Many of these decisions are taken in the *cis*-regulatory regions of genes, which can function as analog implementations of logic gates [1–3]. A classical example is the lactose system in the bacterium Escherichia coli, where the *lac* operon is strongly expressed only if the concentration of active CRP, due to the absence of glucose, is high and that of active LacI, due to the presence of lactose, is low. This network can be interpreted as a logic gate with two input signals, namely the concentrations of the transcription factors (TFs) CRP and LacI, and one output signal, the expression level of the operon; indeed, this gate could be classified as an ANDN gate. The lactose system has been studied in much detail both theoretically and experimentally and is now fairly well understood [4–7]. However, even in prokaryotes, many *cis*-regulatory regions are much more complex than that of the *lac* operon. Figure 1, taken directly from the EcoCyc database version 9.5 [8], shows four typical examples. The *cis*-regulatory regions often contain long tandem arrays of TF binding sites. Moreover, many TFs can both activate and repress the same operon. Perhaps most strikingly, TF binding sites often overlap with one another. We have performed a statistical analysis of the importance of repetitive and overlapping binding sites in *E. coli,* based on the EcoCyc database [8]. The results are shown in Figure 2. We find that 37% of the TF–operon interactions are mediated by more than one binding site and 39% of the binding sites overlap with at least one other site. The question arises what kind of functionality these complex structures can convey [9]. Here we present theoretical results that suggest that these intricate structures are a consequence of the functional requirement of *cis*-regulatory domains to integrate signals. Our results identify a new mechanism for signal integration during transcriptional regulatory control, which is based upon the interplay between cooperative binding of TFs to adjacent sites and competitive binding of TFs to overlapping sites.

To elucidate the origin of the complicated structures shown in Figure 1, we have adopted a novel approach. Using an evolutionary algorithm [10], we have designed prokaryotic *cis*-regulatory domains with predefined functions in silico. In our approach, no specific promoter architectures are specified a priori: the space of possible architectures is sampled in an unbiased manner. This makes it possible to elucidate new architectures and to find the optimal design for a *cis*-regulatory domain that is consistent with a required function. The design principles of these architectures are then extracted a posteriori. As we will show below, this approach has allowed us to reveal new design principles of transcriptional regulation, which would have been difficult to obtain using the more conventional approach of studying particular architectures.

**Figure 3:**
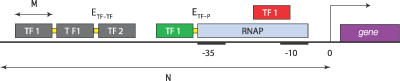
Illustration of the Model The *cis*-regulatory region consists of *N* = 100 bp directly upstream of the transcription start site. In *E. coli,* most TFs bind to this region, although binding sites are also found downstream of the transcription start site; mechanisms requiring such downstream sites are excluded by our model. A TF binding domain counts *M* amino acids, which can bind *M* = 10 bp [54,55]. When two TFs bind within a distance less than *k* = 3 bp, they interact with energy *E*_TF−TF_; this is indicated by a yellow connection between the TFs, although it should be realized that these cooperative interactions could also be mediated via the DNA. When a TF binds close to the RNAP, we assume an interaction energy *E*_TF−P_. The core promoter, consisting of the −10 and −35 hexamers, is indicated; when the RNAP binds to it, it blocks both hexamers and the spacer between them. The TF that binds overlapping with the RNAP is red, to indicate that it represses transcription by steric hindrance; the green TF is an activator, since it recruits RNAP. The gray TFs bind too far upstream from the core promoter to influence the transcription rate. In our simulations, we used *k* = 3 and *E*_TF−TF_ *= E*_TF−P_ = 3.40 *k_B_T* or 2.0 kCal/mol (so that *e* ^β*E*_TF−TF_^ = 30.0) [= 30 [1].

To design prokaryotic *cis*-regulatory domains, we have developed a new model of prokaryotic transcriptional regulation, in which the input–output relation of an operon is deduced from the amino-acid sequences of the TFs and the base-pair (bp) sequence of the *cis*-regulatory region of the operon. To go from sequence to network function, i.e., the input–output relation, the model contains the following key ingredients (see Figure 3): (i) each TF can bind anywhere on the *cis*-regulatory region; this directly implies that to a given location, all TFs can bind; (ii) the affinity of a TF for a certain location is determined by its DNA sequence and the amino acids in the DNA-binding domain of the TF; the binding energies of the amino-acid–bp contacts are extracted from a matrix that is based on crystallographically solved protein–DNA complexes [1]; (iii) TFs cannot overlap in space, but binding sites can overlap along the DNA; TFs thus compete with each other for binding to overlapping sites; (iv) TFs that bind close to each other on the DNA exhibit a cooperative interaction [6]; we consider the case where a TF can bind cooperatively with two neighboring TFs, thus allowing for oligomerisation on the DNA; although some TFs are known to have this property, this is not likely to be a generic property of all TFs; (v) the transcription rate of operons is controlled via the mechanism of “regulated recruitment,” meaning that TFs function by stimulating or hindering the binding of RNA polymerase (RNAP) to the DNA [6]. Although this is the dominant mechanism in prokaryotes, we note that many alternative mechanisms are used as well (see Text S1). To describe the input–output relationship for an operon quantitatively, we employ the statistical mechanical approach developed by Shea and Ackers [12] and Buchler et al*.* [1].

This model makes it possible to design *cis*-regulatory domains by performing rounds of mutation and selection in an evolutionary algorithm. Because the input–output relation is completely specified at the microscopic level of the amino-acid sequences of the TFs and the bp sequences of the *cis*-regulatory regions, new architectures can be obtained by introducing mutations at the microscopic (sequence) level, while selecting at the macroscopic level of the input–output relation. Importantly, neither the architectures of the *cis*-regulatory regions, nor the functional form of the gene regulatory functions, have to be specified a priori: in the course of our simulations, TF binding sites emerge naturally as sites with a particularly high affinity for a certain TF. While the evolutionary algorithm is not designed to closely mimic natural or directed evolution, it does make it possible to freely explore the space of possible promoter architectures.

**Figure 4:**
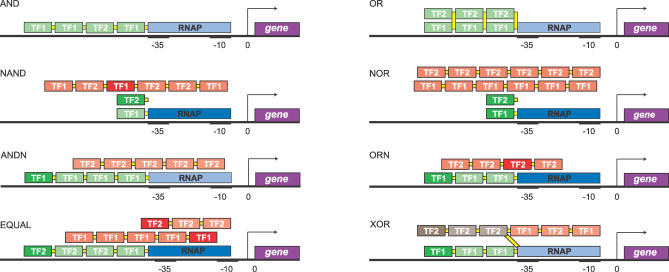
Cartoons of *cis*-Regulatory Constructs Emerging from Our In Silico Design of Transcriptional Logic Gates The boxes indicate the TF binding sites; green indicates that a TF acts as an activator, red that it acts as a repressor, and brown that the action of the TF depends upon the concentrations of the two TFs. Weak binding sites (*K*_D_ > 2 × 10^3^ nM) have a light color, strong ones are dark. Yellow connections between TFs signify cooperative interactions. The designs show that the logic gates are constructed as overlapping arrays of cooperative binding sites. Each layer acts as a module, either activating or repressing transcription. Signals are integrated via the interplay between intramodular cooperativity and intermodular competition.

We have used our approach to design all possible transcriptional logic gates with two input signals and one output signal (see Table 1). These gates have been studied by Buchler et al. using a rational design approach [1]. Our simulations, however, unravel new design principles. In spite of the simplicity of the model, quite complex functionality can emerge. In particular, we find that promoter architectures are often constructed from modules that consist of tandem arrays of binding sites to which TFs can bind cooperatively (see Figure 4). Furthermore, these modules often overlap, leading to competition between them. We show that the intricate interplay between intramodular cooperativity and intermodular competition allows for a wide range of regulatory functions.

**Table 1:**
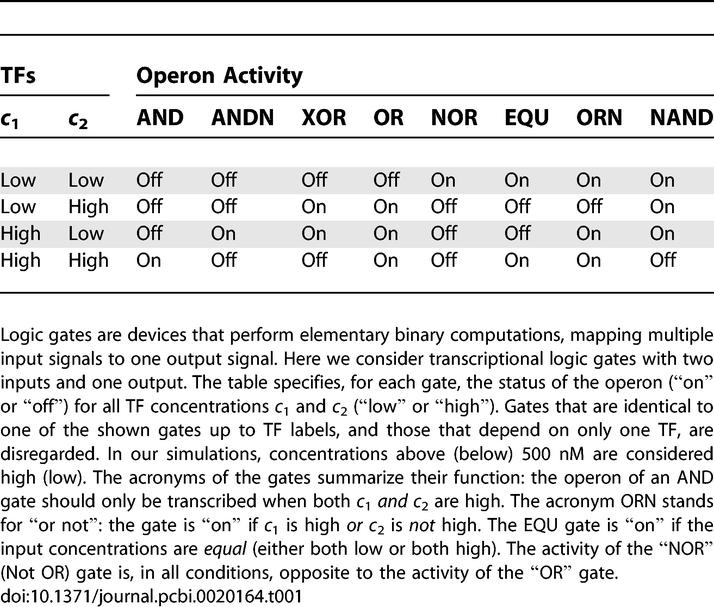
Truth Tables of Transcriptional Logic Gates

## Methods

In the next section, we describe our model of prokaryotic transcriptional regulation, in which the input–output relation is determined by the amino-acid sequences of the TFs and the bp sequence of the *cis*-regulatory region of the operon. The evolutionary design method, in which mutations are made at the level of the TFs' amino-acid sequences and the promoter's bp sequence, while selection is performed on the input–output relation, is described in the subsequent section.

### Model of Transcriptional Regulation

We assume that the transcription rate of an operon is proportional to the fraction of time RNAP is bound to the promoter [1,12–14]. The model we use to compute this quantity is illustrated in Figure 3. The RNAP-σ binds only to the −10 and −35 hexamers, called the core promoter, and we determine its binding energy by comparing the core promoter to a large set of real E. coli promoters [15–18] (see Text S1). We ignore the fact that, in some promoters, the affinity of the RNAP for the promoter is enhanced by interactions of its *α* C-terminal domain with DNA upstream of the −35 hexamers. TFs can bind to any site in the *cis*-regulatory region. Whenever a TF binds to the DNA, each amino acid interacts with exactly 1 bp, and the total binding free energy is the sum of the contributions of each amino-acid–bp contact. This is known to be a reasonable approximation for many TFs, although exceptions have also been documented [16–21]. The binding energies associated with each amino-acid–bp contact are extracted from a matrix based on crystallographically solved protein–DNA complexes [1]. The results, however, do not depend critically upon the precise values of the matrix elements; random matrices with the same mean and standard deviation give similar results. Note that some real TFs can bind ligands or can become phosphorylated; in that case the TF concentration in our model corresponds to the concentration of the DNA-binding form of the TF.

The model allows for two types of TF–TF and TF–RNAP interactions (see Figure 3) [6]. First, we include steric hindrance: molecules cannot overlap in space. Second, we include a cooperative interaction of energy *E*_TF−TF_ between any pair of TFs when they bind within a distance of *k* bp. Likewise, if a TF and RNAP bind close together, we assume a synergetic energy *E*_TF−P_ [24]. We thus assume that in our model TFs can bind cooperatively with themselves, with RNAP, and with other TFs. Although some TFs are known to have all these properties (for instance MalT and MelR), it is unlikely that this is the case for all TFs. Our results will show, however, that combinations of some of these properties allow for myriad promoter functions.

Cooperative interactions between proteins can have two distinct origins. The first is via direct contact between patches on their surface. On the better-characterized, but relatively simple promoters, TFs typically exhibit cooperative interactions with one adjacent TF on the DNA, thus leading to dimers, and not to longer oligomers. Nevertheless, experiments show that TFs exist that bind cooperatively to multiple binding sites (e.g., MalT [25], MelR [26], MetJ [27], Lrp [28], Fur [29], and ArcA [30,31]). Indeed, complex promoters with long arrays of binding sites are frequently observed, as shown in Figures 1 and 2. It is conceivable that on these complex promoters the TFs have multiple patches, thus allowing them to bind cooperatively into long oligomers. In this context, it is important to note that these protein–protein interactions are very weak and are therefore not likely to be detected in large-scale experiments such as those of [32].

The second mechanism for protein–protein interactions is indirect. Here, the interactions are mediated via the DNA. Cooperativity can result from bending, stretching, or super-coiling the DNA by one of the molecules, thereby affecting the binding affinity of the other [6,33]. Although the nature and the strength of these cooperative interactions is still not fully understood, at the level of our statistical–mechanical model, such mechanisms can be described in the same way as cooperativity by direct contact. This means that most effects of *local* chromosome structure are implicitly included in the model. Importantly, such indirect interactions could also give rise to TFs binding cooperatively into long oligomers.

However, the model does not allow action at a distance. Therefore, mechanisms involving *global* chromosome structure, such as DNA looping, are not included. Also, mechanisms that rely on direct interactions between the RNAP and TFs bound farther upstream, for instance, through contact with the flexible RNAP *α* C-terminal domain, are not possible in our model, although it could be extended to incorporate such effects [1].

We use the statistical mechanical approach developed by Shea and Ackers [12] and Buchler et al*.* [1] to describe the input–output relationship for an operon in a quantitative way. To compute the influence of each TF on the transcription rate in a tractable way, we have developed a fast algorithm that efficiently takes into account all TF–DNA, TF–TF, and TF–RNAP interactions (see Text S1).

### Evolutionary Design of Logic Gates

We combined our model with an evolutionary algorithm to design transcriptional logic gates consisting of one operon, regulated by two TFs. Typically, 250 gates, with initially random DNA and amino-acid sequences, were subjected to cycles of mutation and selection. In each cycle, point mutations were introduced; the probability of a mutation occurring within a given *cis*-regulatory region or TF was 0.85 and 0.3, respectively, but the results do not depend strongly on these values. Next, the top 20% of the gates were selected and the others were removed. To complete the cycle, we finally refilled the empty slots by copying randomly chosen genotypes from the selected gates.

**Figure 5:**
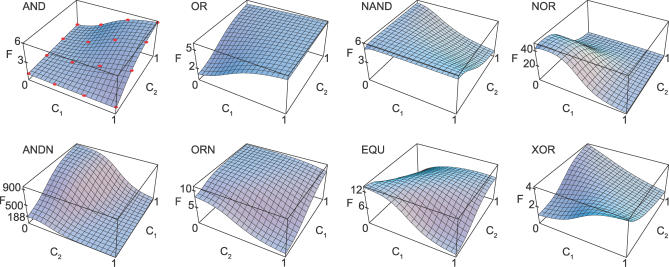
Response Plots of Logic Gates Emerging from the Simulations The quantity *F* on the vertical axis is the fold change of the transcription rate, defined here as *F* = *A*(*c*_1_,*c*_2_)/*A*_min_, where *A*_min_ is the minimal transcription rate on this TF concentration domain. The concentrations *c*_1_ and *c*_2_ are in *μ*M and plotted on a linear rather than a logarithmic scale. The red dots in the AND gate indicate the measurement points (*c*_1_,*c*_2_) that are used in the fitness function.

To select the top 20% of the gates, we define a fitness function that quantifies the quality of the gate. The transcription rate *A* of a gate depends on the concentrations *c*_1_ and *c*_2_ of the two TFs: *A* = *A*(*c*_1_,*c*_2_). First, we compute the transcription rate for 16 values of (*c*_1_,*c*_2_) in the range 0–1,000 nM; for the AND gate in Figure 5, these 4 × 4 values are depicted as red dots. For each of these points, we determine how far *A* deviates from a goal function *G*(*c*_1_,*c*_2_), which is defined by the logic gate we are trying to obtain. Next, we compute the sum of the squares of these deviations. If this quantity is small, then the fitness is considered high (see Text S1). Our fitness function selects for rather steeply switching gates, since the switching is required to take place between *c_i_* = 333 nM (considered low) and *c_i_* = 667 nM (considered high). We also implicitly assume that all conditions are equally important; each of the 16 points has an equal weight in the fitness function. In reality, this is not necessarily the case: the fitness cost of a gene being “on” at a wrong time, need not match the cost of one that is “off” when it should not be (see also [33]). To elucidate general design principles, we select for idealized promoter functions, although, clearly, in nature the input–output relations can be more intricate; an example is the *lac* promoter, which is not a perfect ANDN gate [7].

## Results

### *cis*-Regulatory Constructs

**Figure 6:**
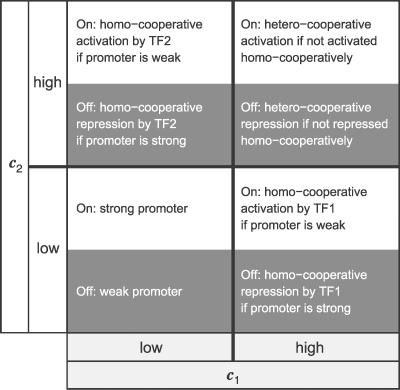
Table Summarizing Which Homo-Cooperative or Hetero-Cooperative Activation or Repression Modules Are Needed to Obtain a Particular Transcriptional Logic Gate The table consists of four quadrants, corresponding to different TF concentrations *c*_1_ and *c*_2_ (each being low or high). Each quadrant is divided into two parts (white and gray), corresponding to the alternative promoter states (on or off). As an example, the AND gate is on only if both TF1 and TF2 are present; this requires a hetero-cooperative activation module. In contrast, an OR gate should be on if either TF1 or TF2 is present. This requires homo-cooperative activation modules for each of the species, because the promoter is weak (the gate must be off when both species are absent); however, since the activation modules do not compete with one another, a hetero-activation module is not required: the homo-cooperative activation modules also turn the gate on when both TFs are present. In general, the design can be most easily understood by first considering the design constraints when both TFs are absent, then the requirements when one of the two are present, and lastly the design constraints when both TFs are present. The EQU and XOR gates discussed in the main text illustrate this perhaps most clearly. Note that the EQU gate is an example of a gate in which a hetero-activation module is required, despite the fact that the promoter is strong; the hetero-activation module is needed to counteract the two homo-cooperative repression modules when both TFs are present.

Figures 5 and 6 show typical simulation results for the gates in Table 1. Clearly, the architectures can be quite complex. Interestingly, the final constructs do not depend much on the initial conditions; this can be regarded as a simple example of convergent evolution. Moreover, they are remarkably similar to the structures found in *E. coli,* as we now describe.

### Homo-Cooperative Auxiliary Sites Provide Steep Responses

We can distinguish two kinds of binding sites. Binding sites from where the TFs directly interact with the RNAP are called *primary* sites. Primary activator sites are located right next to the −35 hexamer of the core promoter, while primary repressor sites directly overlap with the core promoter. The remaining binding sites are called *auxiliary* or *secondary* sites [9]. These sites provide cooperativity. The main function of cooperativity between identical TFs, called *homo*-cooperativity, is to create steep responses [1,34]. We find that activating and repressing binding sites are both regularly supported by (tandem arrays of) auxiliary sites.

#### Activation.

In cooperative arrays of activation sites, the auxiliary site farthest removed from the core promoter usually has the strongest affinity. This can be seen in the *cis*-regulatory regions of EQU, ORN, XOR, and ANDN. Further analysis shows that this pattern enhances the steepness of response (see Text S1). The steepness is optimal if the binding affinities of the farthest site and those of the other sites differ by a factor of 2 to 14, depending on the strength of the promoter, the value of the interaction energies (*E*_TF−P_ and *E*_TF−TF_), and the number of tandem repeats: this way, the steepness can be enhanced up to 27%. A similar result was presented in [14] for systems with one auxiliary site, in the context of the regulation of the phage λ promoter *P*_RM_. We therefore predict that activating auxiliary sites in real promoters regularly have a higher affinity than their primary sites.

It may be useful to repeat that we define auxiliary sites as sites that do not interact directly with the RNAP. If, in real E. coli promoters, one of the upstream sites does interact with RNAP, for instance via direct contact with the α C-terminal subdomain of the RNAP, then such a site is, by definition, a primary site. If such a distant primary site is accompanied by an auxiliary site, then this auxiliary site still needs to have a higher affinity than its primary site, in order to maximize the steepness of response.

In *E. coli,* homo-cooperative activation occurs regularly. For example, the TFs of the LysR family often bind to two sites, one at −65 and the other close to the −35 hexamer of the core promoter [35,36]. In some cases, the TFs bind cooperatively to these sites; in these cases the site at −65 has a stronger affinity than that near −35 [37,38], as one would expect from our results. Another example is the activation of the *P*_RM_ promoter in phage-λ by CI, which binds more strongly to the auxiliary site (O_R1_) than to the primary activation site (O_R2_) [12,14]. We note however, that this example is complicated by the fact that O_R1_ and O_R2_ are also involved in repressing the *P*_R_ promoter. We will get back to this in the next subsection.

#### Repression.

In contrast to the activation modules, the auxiliary sites in repressor complexes are usually much *weaker* than the primary ones (see, e.g., ORN and EQU). Further analysis (see Text S1) shows that the steepness of repression is optimal if the primary site has a 5× to 50× higher affinity than the auxiliary sites (depending on the promoter strength, the values of the interaction energies, and the number of tandem repeats). This pattern can increase the maximal steepness of the response by about 70%, as compared with the case where all sites have an equal affinity. We therefore predict that auxiliary sites in real repressor systems should often be weak.

Indeed, most well-characterized repressor systems in E. coli have auxiliary operators [9,39], many of which are weak. For example, the two cooperative Fur-binding sites that overlap the core promoter on the pColV-K30 plasmid are supported by an array of low-affinity auxiliary sites [29]. A second example is the duo of *dnaA* promoters, 1P and 2P [40]. At low concentrations, DnaA represses only 1P, but at high concentrations it blocks both promoters, as a result of the cooperative binding of up to four DnaA monomers to weak binding sites overlapping the 2P region [40]. Other examples are the TrpR repressor on the *trp* promoter [41] and the Fis repressor on the *aldB* promoter [42]. Finally, the *gltA-sdhC* intergenic region contains at least two high-affinity ArcA-P repressor sites, one overlapping the *gltA* promoter and one blocking the *sdhC* promoter; at higher ArcA-P concentrations, both binding regions broaden until ArcA-P covers a region of about 230 bp, suggesting ArcA-P oligomerization on the DNA [30,31].

In the previous subsection, we mentioned the activation of *P*_RM_ by CI in the bacteriophage λ as an example of cooperative activation, and argued that steep activation requires that the auxiliary site O_R1_ should be considerably stronger than the primary site O_R2_. Interestingly, the same CI binding sites O_R1_ and O_R2_ are also involved in repressing the *P*_R_ promoter. But the binding sites now have reversed roles: from the point of view of promoter *P*_R_, O_R1_ is the primary repressor site, and O_R2_ is auxiliary. However, since we just concluded that, in repressor systems, primary sites should be stronger than auxiliary sites, we conclude that both for steep activation of *P*_RM_ and for steep repression of *P*_R_, site O_R1_ needs to be stronger than site O_R2_, as is indeed the case.

As a final remark on homo-cooperativity, we point out that, while cooperativity is used widely, as Figure 1 shows, many of the better-characterized promoters, such as the *lac* promoter, have a simpler architecture. It should be realized that the number of binding sites not only depends upon the complexity of the desired input–output relation, but also upon the required cooperativity. If, for instance, we select for simpler gates with a weaker response function, we do obtain simpler promoter architectures (unpublished data).

### Hetero-Cooperativity Provides Conditional Responses

While the benefit of homo-cooperativity is to create steep responses, the function of cooperativity between *different* molecular species, *hetero*-cooperativity, is rather to integrate signals. It can be used whenever a response should be conditional on the presence of more than one TF. A good example is the AND gate. As with the OR gate, this gate requires a weak promoter—this ensures that the operon is not transcribed when both TFs are absent. In contrast to the OR gate, however, the AND gate should be on only when both TF1 and TF2 are present. The activation is therefore mediated by a TF1 binding site that is too weak to be functional by itself. Next to this site, a stronger TF2 binding site is present. Only when TF1 and TF2 are both present do they bind cooperatively and induce activation [1]. The remaining sites can bind either TF1 or TF2 and are responsible for the steepness of the response.

#### Activation.

Hetero-cooperative activation is found regularly in naturally occurring promoters. A good example is the activation of the *melAB* operon by MelR, which binds to four sites [25,26]. A CRP binding site is present between MelR sites 2 and 3. Here, CRP binds cooperatively with the downstream MelR sites. This increases their fractional occupancy, resulting in transcription activation. Another excellent example is the *malKp* promoter (see Figure 1D) [25,43], which is discussed below.

#### Repression.

The CytR regulon provides an example of hetero-cooperative repression. CytR often binds cooperatively with cAMP-CRP to form a repression complex. Good examples are *udp* [44], *nupG* [45], *tsx-p2* [46] and *deoP2* [47]; see also [48,49]. Recently, it has also been shown that Lrp and H-NS act cooperatively at the *rrnB* promoter [50].

### Competition between Modules

Whenever binding sites overlap, competition between TF complexes occurs. It is well-known that the core promoter often overlaps with an operator; this is a standard repression mechanism [9]. The role of overlapping TF binding sites in signal integration has been less commented on. Clearly, a repressor that binds to an operator overlapping with an activator site can be used to create anti-activation. Likewise, anti-repression occurs when a binding site overlaps with a repressor site, but not with the core promoter. But the full potential of this type of competition only becomes clear when it is combined with cooperativity. Our NOR, NAND, EQU, and XOR gates serve as instructive examples.

#### Sharpening repression by competitive activation.

The NOR gate (see Figures 5 and 6 and Table 1) combines competition and homo-cooperativity. This gate contains both activator and repressor sites for each TF. The single activator sites are strong compared with the repressor sites; as a result, activation dominates at low TF concentrations. However, as the TF concentrations increase, the affinity of the repressor module grows more rapidly; this is the result of the homo-cooperativity between the repressor sites. Consequently, at high TF concentrations repression dominates. The function of the activating sites is thus to counteract repression at low concentrations, thereby increasing the switching steepness. As it turns out, whenever we select for steep repression, we also get activation. The general message is that using competing modules containing different numbers of homo-cooperative binding sites, a TF can effectively be both an activator and a repressor, depending on its concentration.

The NAND gate looks rather similar to the NOR gate, but uses hetero- instead of homo-cooperativity. Repression dominates only if both TF1 and TF2 are present in sufficient concentrations. This shows that by combining competition and hetero-cooperativity, a TF can either be an activator or a repressor, *conditionally* on the concentration of another TF.

#### Intramodular cooperativity and intermodular competition.

In the EQU gate all mechanisms act in concert. In an EQU gate the operon must be on when the concentrations of both TFs are low; this requires a strong promoter. If either TF1 or TF2 is present, the operon must be off; this requires homo-cooperative repression modules, which block the binding of RNAP when either TF1 or TF2 is present. However, if both TF1 and TF2 are present in similar concentrations, the operon must be on; this requires a hetero-cooperative activation module that counteracts the effect of the homo-cooperative repression modules.

In the XOR gate, the same mechanisms act, but in an opposite manner: if both TFs are absent, the operon should be off; this requires a weak promoter. If one of the two TFs is present, the operon should be on; this demands homo-cooperative activation modules, which recruit the RNAP when only one of the two TFs is present. If both TFs are present, however, the operon should be off; this requires a hetero-cooperative repression module that neutralizes the actions of the homo-cooperative modules when both TFs are present.

In both gates, the homo-cooperative and hetero-cooperative modules have to compete with one another. This is achieved via the binding of the TFs to overlapping binding sites. Which module wins the competition depends upon the TF concentrations, the number of TFs in the modules, and upon the quantitative details of the protein–protein and protein–DNA interactions. Text S1 discusses both gates quantitatively.

Similar mechanisms are known to occur in E. coli. The *malKp* promoter (see Figure 1D) provides a good example, although its full input–output relation is more complex than those of the logic gates studied here. In the presence of CRP, MalT binds to three tandem sites to form the activation complex [25,43]. In the absence of CRP, however, MalT binds with relatively high affinity to an alternative triplet of repressor sites that overlaps the activation complex, thereby repressing *malK*. As in the EQU gate presented here, the activation complex has to compete with the repression complex; the CRP concentration determines whether MalT acts as a repressor or as an activator [25,43].

## Discussion

We have developed a model of transcriptional regulation and applied it to the evolutionary design of transcriptional logic gates in prokaryotes. Our approach has revealed new design principles, which would have been difficult to predict using a rational design approach. In particular, our analysis stresses the importance of the interplay of the following mechanisms: 1) homo-cooperative interactions between TFs within modules; 2) hetero-cooperative interactions between TFs within modules; 3) competition between TF modules. Using these mechanisms only, a wide range of input–output relations can be produced, including the full repertoire of *cis*-regulatory logic gates with two input signals and one output signal.

The resulting constructs make extensive use of cooperative tandem binding sites. Homo-cooperativity is often used as a means of achieving high Hill coefficients. In such tandem arrays of binding sites, weak sites can be important. In repressive arrays, auxiliary sites are usually weak, while in activating arrays the auxiliary sites tend to have the highest affinity. Hetero-cooperativity allows for regulation conditional on the presence of more than one TF species. Hetero-cooperativity within modules thus plays a central role in integrating different signals; in the gates studied here, a hetero-cooperative module only becomes active if both TFs are present. While many promoters in nature exhibit long arrays of binding sites (see Figures 1 and 2), it seems unlikely that all TFs of E. coli have the capacity to bind cooperatively into long arrays. Indeed, the origin and the degree of cooperativity in these complex structures is still far from understood. We hope that our simulation results encourage experimentalists to characterize complex promoter architectures in more detail.

The capacity to integrate signals is dramatically enhanced by the competition between different modules, as summarized in Figure 6. Competing modules allow the integration of signals, because a) both homo- and hetero-cooperative modules can act as activator modules or as repressor modules; b) when the concentrations of the TFs change, the relative activities of the activating and repressing modules also change. How their activities change with the TF concentrations depends upon the strength of the TF–DNA, TF–TF, and TF–RNAP interactions. It also depends upon the degree of cooperativity: the number of binding sites in a module not only determines the steepness of the response, but also affects the concentration range in which the module is active—a large module will dominate an overlapping, but smaller one at sufficiently high TF concentrations, even when the individual TFs in the larger module have a weaker affinity for the DNA. Indeed, not only hetero-cooperativity but also homo-cooperativity can play an essential role in signal integration (see also Figure 3 in Text S1). In Text S1, we discuss in more detail how the mechanisms of cooperative and competitive binding of TFs could be used for the rational design of transcriptional logic gates.

Our results provide a possible explanation for the complexity of *cis*-regulatory regions found in *E. coli,* which, indeed, often contain tandem TF binding sites and overlapping sites. Our analysis suggests that these complex architectures are a natural consequence of the basic mechanisms of transcriptional regulation and, on the other hand, the function of *cis*-regulatory domains to integrate signals. While we focus here on prokaryotes, it should be clear that similar integration mechanisms might also operate in the *cis*-regulatory domains of transcription units in eukaryotes; ample anecdotal evidence exists, e.g., for the role of adjacent and overlapping TF binding sites in signal integration during embryonic development of the sea urchin [3] and *Drosophila* [51]. Our results also emphasize that understanding the complex promoters observed both in our simulations and in nature, requires quantitative knowledge of binding affinities and interactions: from the binding site locations only, it is often not possible to distinguish an AND gate from an OR, nor a NAND from a NOR.

In this paper, we have used our evolutionary design method to design *cis*-regulatory domains of single operons. This method, however, could also be applied to design larger networks, such as multi-input modules [52]. As the network size increases and regulons become larger, we expect that it will become increasingly more difficult to fulfill all constraints imposed on the promoter and TF sequences. For these larger networks, not only *positive* design—selecting for desired TF–DNA interactions—but also *negative* design—selecting against unwanted TF–DNA interactions—may be an important design criterion. Our approach could also be extended to design feedback networks. By selecting transcription networks containing multiple genes based on their *dynamics,* we can design feedback systems such as transcriptional oscillators and bistable switches [10].

Here, we used our method to design transcriptional logic gates. For this reason, our evolutionary algorithm was not developed to mimic natural or directed evolution. However, with suitable modifications and extensions, our approach could also be used to study questions that are pertinent to the evolution of functional promoter regions, such as what the pathways of evolution are, and how the evolution of logic gates depends upon factors such as population size, neutral drift, and mutation rates.

Finally, the proposed signal integration mechanism of intramodular cooperativity versus intermodular competition could be tested experimentally by rationally designing *cis*-regulatory constructs. But perhaps more interesting would be to see whether an evolutionary design method can be used. Recently, Yokobayashi et al. demonstrated experimentally that directed evolution can be used to change protein–DNA and protein–protein interactions in a rationally designed, but nonfunctional gene circuit to obtain a functional network [53]. Perhaps a similar method can be used to design, by experiment, transcriptional logic gates with desired input–output relations. Since no specific promoter designs have to be imposed, it would be interesting to see whether the resulting architectures exploit the signal integration mechanism of competing binding site modules.

## Supporting Information

Text S1 Supplementary Information (1.7 MB PDF)

### Accession Numbers

In Table 2 we list SwissProt database accession numbers of the genes and proteins mentioned in this article.

**Table 2:**
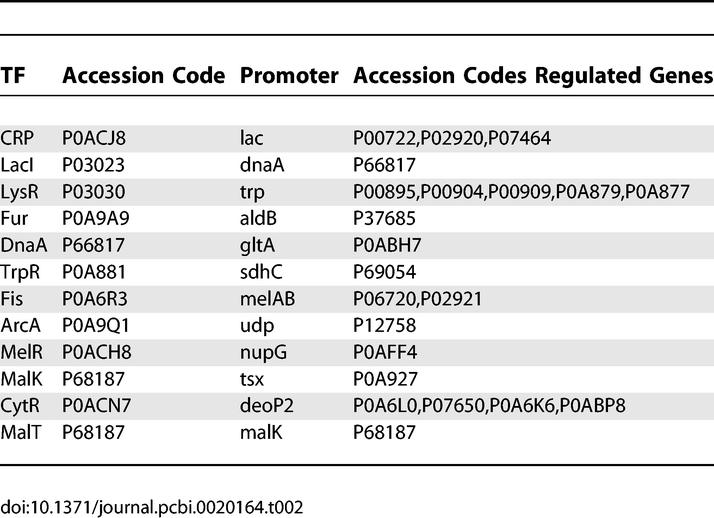
SwissProt Accession Codes for Genes and Proteins Mentioned in This Article

## Abbreviations

RNAP: RNA polymerase
TF: transcription factor
