# Supplementary material for: Transcriptional Regulation by Competing Transcription Factor Modules
Source: PLoS Comput Biol. 2006 Dec 1;2(12):e164. doi: 10.1371/journal.pcbi.0020164 (PMC1676028; doi:10.1371/journal.pcbi.0020164)
Supplement: Supplementary file 1 [file pcbi.0020164.sd001.pdf]

# Supporting Text S1

Supplementary Information for

*Transcriptional Regulation by Competing Transcription Factor Modules*

Rutger Hermsen, Sander Tans, Pieter Rein ten Wolde

## I. DETAILED DESCRIPTION OF THE MODEL

Our model needs to address four quantities: the binding affinities of each transcription factor (TF) for every possible site on the *cis*-regulatory region, the affinity of RNAP-$\sigma$ for the core promoter, the interactions between the molecules, and finally the transcription rates based on these affinities and interactions. We discuss each of these issues below.

### A. Binding of TFs to DNA

TFs can bind anywhere on the *cis*-regulatory region. The affinity of a TF for a given site is determined by the DNA sequence at the site and the amino-acid sequence in the DNA binding pocket of the TF. We assume that, whenever a TF $a$ binds to a binding site $O$, each amino acid interacts with exactly one base pair, and that the total binding free energy $E_{a,O}$ is the sum of the contributions of each amino-acid - base-pair contact. This means that the binding free energy of a TF $a$ with amino acids $a_i$ to a binding site $O$ with base pairs $O_i$ is given by

$$E_{a,O} = \sum_{i=1}^{M} U_{a_i,O_i}.$$

Here $U_{\lambda,\mu}$ is a $20 \times 4$ matrix containing the binding free energies associated with each amino-acid - base-pair contact. We used a matrix given in reference [1], based on christallographically solved protein-DNA complexes.

Finally, the binding affinity $q_{a,O}$ of TF $a$ for site $O$ follows from

$$K_{a,O} = \alpha e^{-\beta E_{a,O}}, \quad (1)$$

$$q_{a,O} = \frac{c_a}{K_{a,O}}. \quad (2)$$

Here, $K_{a,O}$ denotes the dissociation constant and $c_a$ denotes the concentration of TF $a$. The proportionality factor $\alpha$ in equation 1 is determined by the free energy of all other sites that compete with $O$ for binding of the TF. We used $\alpha = 10^7$nM, but also found that the results do not depend critically on this value.

### B. Binding of RNAP

In our model, the RNAP-$\sigma$ complex binds only to the core promoter. We determine the binding free energy of RNAP-$\sigma$ for a core promoter $p$ by comparing the -10 and -35 hexamers to a large set of real *E.coli* promoters, taken from reference [2]. To every base pair $p_i$ at position $i$ within the $-10$ and $-35$ hexamers, we assign a score $s_i$; it equals the fraction of real *E.coli* promoters that have $p_i$ at that particular position, normalized by the random fraction 0.25. Next, the binding energy $E_p$ of the RNAP to that particular core promoter can be estimated by [3–5]:

$$E_p = k_B T \sum_{i \in p} \log(s_i).$$

The dissociation constant of the binding reaction, $K_p$, and the binding affinity of the RNAP for the promoter, $q_p$, now follow from the equations

$$K_p = \alpha' e^{-\beta E_p}, \quad (3)$$

$$q_p = \frac{c_p}{K_p} \propto c_p \prod_{i \in p} s_i. \quad (4)$$

The proportionality factor $\alpha'$ in equation 3 again includes the competition between site $p$ and all other places the RNAP could possibly be; it should be chosen such that $p_{\rm on}$ is close to unity in case of a small number of mismatches, but decreases rapidly as mismatches accumulate. We used $\alpha' = 10^7$nM.

### C. TF-TF and TF-RNAP interactions

The interaction between the molecules consists of two parts. In the first place, we include steric hindrance: TFs and RNAP cannot overlap in space. When bound to the DNA, TFs occupy $M$ base pairs and mutually exclude each other and RNAP. Bound RNAP is assumed to block the consensus hexamers and the spacer in-between. In the second place, we include an unspecific attractive interaction between TFs whenever they bind close to each other – that is, within a distance of $k = 3$ base pairs. To this interaction we associate an energy $E_{\text{TF-TF}}$ of 2 – 4 $k_BT$, such that $\omega \stackrel{\text{def}}{=} \exp(\beta E_{\text{TF-TF}}) \approx 30$ [6]. Likewise, if a TF and RNAP bind close together, we assume a similar interaction free energy $E_{\text{TF-P}}$; Again, $\omega' \stackrel{\text{def}}{=} \exp(\beta E_{\text{TF-P}}) \approx 30$.

### D. Transcription rates

We assume, following Shea and Ackers and Buchler *et al*, that the transcription rate $A$ of an operon is proportional to the fraction of time $p_{\rm on}$ a RNAP is bound to the core promoter [6, 7]. This assumption is reasonable provided the kinetics of the binding and unbinding of RNAP

are sufficiently fast in comparison to the transition rate from the closed to the open complex. In that case the binding reaction is near equilibrium and the fractional occupancy is given by

$$A \propto p_{\rm on} = \frac{Z_{\rm on}}{Z}. \tag{5}$$

Here $Z_{\rm on}$ is the partition sum of all states in which an RNAP molecule is bound, and $Z$ is the total partition sum. This approach is used widely [6–10]. We note however, that this model does not apply to all cases: for instance, some TFs function by regulating the rate of the transcriptional steps after the initial binding of RNAP to the core promoter, and in some cases a tight binding of RNAP to the core promoter might negatively influence the transition rate to the so-called open complex.

### E. Computing the partition sums

In the previous subsections, we explained how to compute the TF binding affinities for each possible position on the *cis*-regulatory region, the affinity of RNAP for the core promoter, and all interaction energies, given the sequences and concentrations of the molecules. This allows us in principle to compute the Boltzmann factor $W(s)$ of every state $s$ and the hence the partition sum $Z$ of the system. But since we assumed that TFs can bind anywhere on the DNA, the total number of states or configurations can easily become huge. In fact, a minimal network consisting of only one operon and two TFs with $N = 80$ and $M = 10$ (see Fig.3 of main text), counts more than three million distinct configurations. We developed a scheme that nevertheless allows us to compute the partition sum for a given promoter in an efficient manner.

We use the following conventions (see Fig.3 of main text). We refer to the stretch of DNA ranging from base pair $i - M + 1$ to base pair $i$ as site $i$. We denote the binding affinity of TF $a$ for site $i$ by $q_{a,i}$. Next we define

$$Q_i \stackrel{\rm def}{=} \sum_a q_{a,i}.$$

Finally we consider a series $Z_i$ of *partial* partition sums ($-N \le i \le 0$), defined as the partition sum of all possible states in which sites with a number bigger than $i$ are not occupied and no RNAP is bound.

Let $s$ be the state where TFs $a_1 \dots a_m$ are bound to sites $x_1 \dots x_m$ respectively. Then in ref. [8] it is explained that the Boltzmann factor $W(s)$ of $s$ equals

$$W(s) = \left(\prod_{u \neq v} \omega_{u,v}\right)\left(\prod_{u=1}^{m} q_{a_u,x_u}\right),$$

where

$$\omega_{u,v} = \begin{cases} \omega & \text{if site } u \text{ and } v \text{ are 0 to } k \text{ bps apart,} \\ 0 & \text{if site } u \text{ and } v \text{ overlap,} \\ 1 & \text{else.} \end{cases}$$

This implies that for the series $Z_i$, the following recurrence relation holds:

$$\begin{aligned} Z_i &= Q_i Z_{i-M-k} + Q_i \omega \left(Z_{i-M} - Z_{i-M-k}\right) + Z_{i-1} \\ &= Q_i \left[(1-\omega) Z_{i-M-k} + \omega Z_{i-M}\right] + Z_{i-1}, \end{aligned} \tag{6}$$

with starting conditions

$$Z_i = \begin{cases} 0 & \text{for } i < -N, \\ 1 & \text{for } -N < i < -N + M. \end{cases}$$

We can express $Z_{\rm off}$, $Z_{\rm on}$ and $p_{\rm on}$ in terms of the $Z_i$ as

$$Z_{\rm off} = Z_0, \tag{7}$$

$$Z_{\rm on} = q_p \left[\omega' Z_x + (1-\omega') Z_{x-k}\right], \tag{8}$$

$$p_{\rm on} = \frac{1}{1 + Z_{\rm off}/Z_{\rm on}}. \tag{9}$$

Here $x$ is the base pair just next to the core promoter ( $x = -37$ ). The conclusion is that, in order to compute $p_{\rm on}$, one only needs to compute the $Q_i$, apply equation 6 $N$ times, and finally fill in expressions 7, 8 and 9. Note that the time required to compute $p_{\rm on}$ using this algorithm scales linearly with $N$, $M$, and the number of TFs. This shows that the scheme is fast and can therefore be applied to networks consisting of many genes and TFs.

## II. FITNESS FUNCTION

In order to select the gates, we need a fitness function that quantifies their quality. We now describe the fitness function we used. The transcription rate $A$ of a gate depends on the concentrations $c_1$ and $c_2$ of the two TFs: $A = A(c_1, c_2)$. We use concentrations in the rage 0 to $10^3$ nM; concentrations below (above) 500 nM are considered low (high). Each truth table $t$ then defines a goal function $G_t(c_1, c_2)$; the perfect analog AND gate, for instance, has the following response:

$$\begin{aligned} A(c_1, c_2) \propto p_{\rm on}(c_1, c_2) &= G_{\rm AND}(c_1, c_2) \\ &= \theta(c_1 - 500{\rm nM})\theta(c_2 - 500{\rm nM}), \end{aligned}$$

where $\theta(x)$ is the Heaviside step function. We define the fitness function $R$ as follows. First, we compute $p_{\rm on}(c_1, c_2)$ for 16 values of $(c_1, c_2)$; for the AND gate in Fig. 5 of the main text, these $4 \times 4$ values are depicted as red dots. For each of those points, we determine how much $p_{\rm on}(c_1, c_2)$ deviates from the goal function $G_t(c_1, c_2)$; next we compute the sum of the squares of these deviations. If this quantity is small, the fitness is considered high. The following equation summarizes the measure:

$$R = -\sum_{i,j=0}^{3} \left[p_{\rm on}\left(\frac{i}{3} \times 500{\rm nM}, \frac{j}{3} \times 500{\rm nM}\right) - G_t\left(\frac{i}{3} \times 500{\rm nM}, \frac{j}{3} \times 500{\rm nM}\right)\right]^2.$$

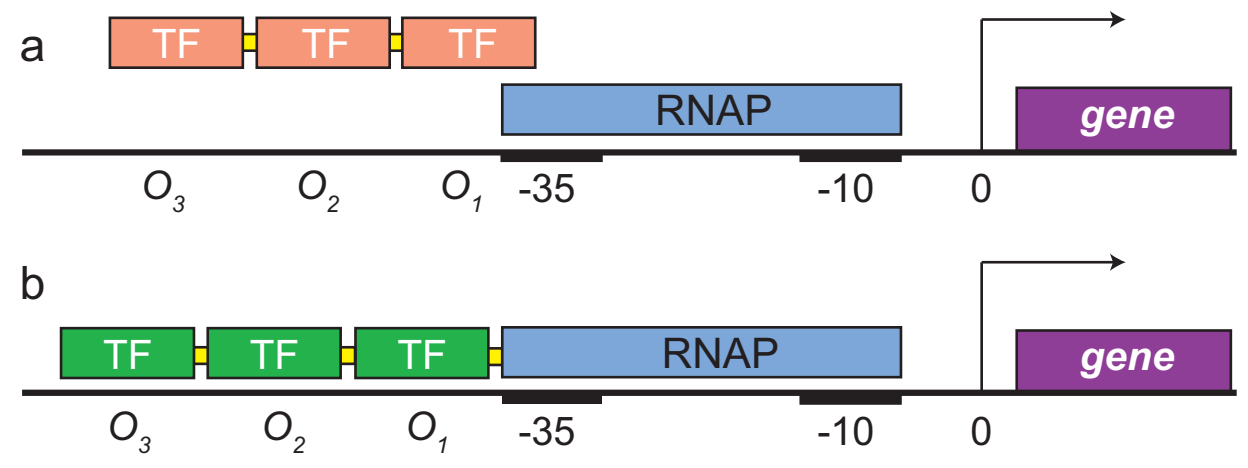


Fig. 1: Illustration of the repression (a) and activation (b) system discussed. In both cases, the TF has three binding sites; $O_1$ is in both cases the primary site, while $O_2$ and $O_3$ are the auxiliary binding sites.

## III. AFFINITIES OF AUXILIARY SITES

One of the main functions of auxiliary binding sites is to create steep responses to changes in TF concentrations. In the results of our simulations, we observed that the auxiliary sites of repressors are often weak, while in case of activator sites they are often strong. Moreover, in activator systems, the auxiliary site furthest removed from the core promoter usually has the highest affinity. Here we demonstrate that these patterns further enhance the steepness of response.

The basic idea is as follows. If the affinity of an auxiliary site is very low, the effect of the site vanishes. On the other hand, if its affinity becomes very large, the auxiliary site will always be occupied. In that case, the auxiliary site merely increases the affinity of the primary site with a constant factor ($\omega$ in our model). The effect of this is equivalent to lowering the dissociation constant of the primary site with the same factor, which shows that in this limit the cooperativity is lost as well. Somewhere between these limits, an optimum must be present. This optimum is different for activating sites and repressing sites.

It is possible to analyse the situation for any number of auxiliary sites. Below we show the results for two auxiliary sites. (See Fig. 1.)

### A. Repression

We assume that a promoter has one primary repressor site and two auxiliary sites (Fig. 1a). The primary repressor site $O_1$ has a dissociation constant $K$, while the auxiliary sites $O_2$ and $O_3$ have dissociation constants $K/r_2$ and $K/r_3$. The question then is: What values of $r_i$ maximize the steepness of the response?

As before, we compute $p_{\rm on}$ according to Eq. 5. The partition sums are:

$$\begin{aligned} z_{\rm on} &= q_p \left(1 + (r_2 + r_3)\left(\frac{c_R}{K}\right) + r_2 r_3 \omega \left(\frac{c_R}{K}\right)^2\right) \\ z_{\rm off} &= 1 + (1 + r_2 + r_3)\left(\frac{c_R}{K}\right) \\ &\quad + (r_2\omega + r_3 + r_2 r_3 \omega)\left(\frac{c_R}{K}\right)^2 \\ &\quad + r_2 r_3 \omega^2 \left(\frac{c_R}{K}\right)^3 . \end{aligned}$$

The concentration of repressor is denoted by $c_R$.

We use three different measures of the steepness of response:

1. We optimized the slope $s_{\rm half}$ of the response plots at the TF concentration at which the expression level is half maximal ($c_{\rm half}$); we choose $K$ such that $c_{\rm half} = 500$ nM. The results are shown in Fig. 2a, where we use $\omega = 50$ and $q_p = 10$. The figure shows that $s_{\rm half}$ can be increased considerably (69%) by optimizing the relative affinities of the auxiliary sites. The best result is obtained at $r_2 = 0.017$ and $r_3 = 0.091$, confirming that, ideally, repressive auxiliary sites are much weaker than their primary sites.

2. We fitted the response plots to Hill functions, defined as

$$H_R(c_R) = A \frac{1 + \left(\frac{c_R}{K}\right)^n / f}{1 + \left(\frac{c_R}{K}\right)^n},$$

where $f$ is the maximum fold-change in expression level and $n$ is the value of the Hill coefficient [6]. We optimized the value of the Hill coefficient $n$ as a function of $r_2$ and $r_3$. The resulting plots (not shown) are very similar to those found using the first method; $n$ can be increased by 63% by choosing $r_2 = 0.011$ and $r_3 = .057$.

3. We optimize the slope $s_{\rm inf}$ at the inflection point of the response curve. Now, we choose $K$ such that this point is at 500 nM. Fig. 2c shows that $s_{\rm inf}$ can be increased by 70% if we fine-tune the affinities of the different binding sites. Again the auxiliary sites are weak: $r_2 = 0.014$ and $r_3 = 0.11$.

All methods show that weak auxiliary sites of repressor systems are not only sufficient, but even optimal. Therefore it is highly unlikely that evolution would maintain strong auxiliary repression sites, if a steep response is beneficial. Of course, this argument only holds for auxiliary sites that do not have a second function. If an auxiliary site also functions as an anti-activator (*i.e.*, it prevents the binding of an activator by overlapping with its binding site) a higher affinity may be required.

Interestingly, our results also show that site 2 should ideally be weaker than site 3. Note, however, that site 3 can be interpreted as an activator site for site 2; this situation is therefore analogous to the activation system, which we discuss below.

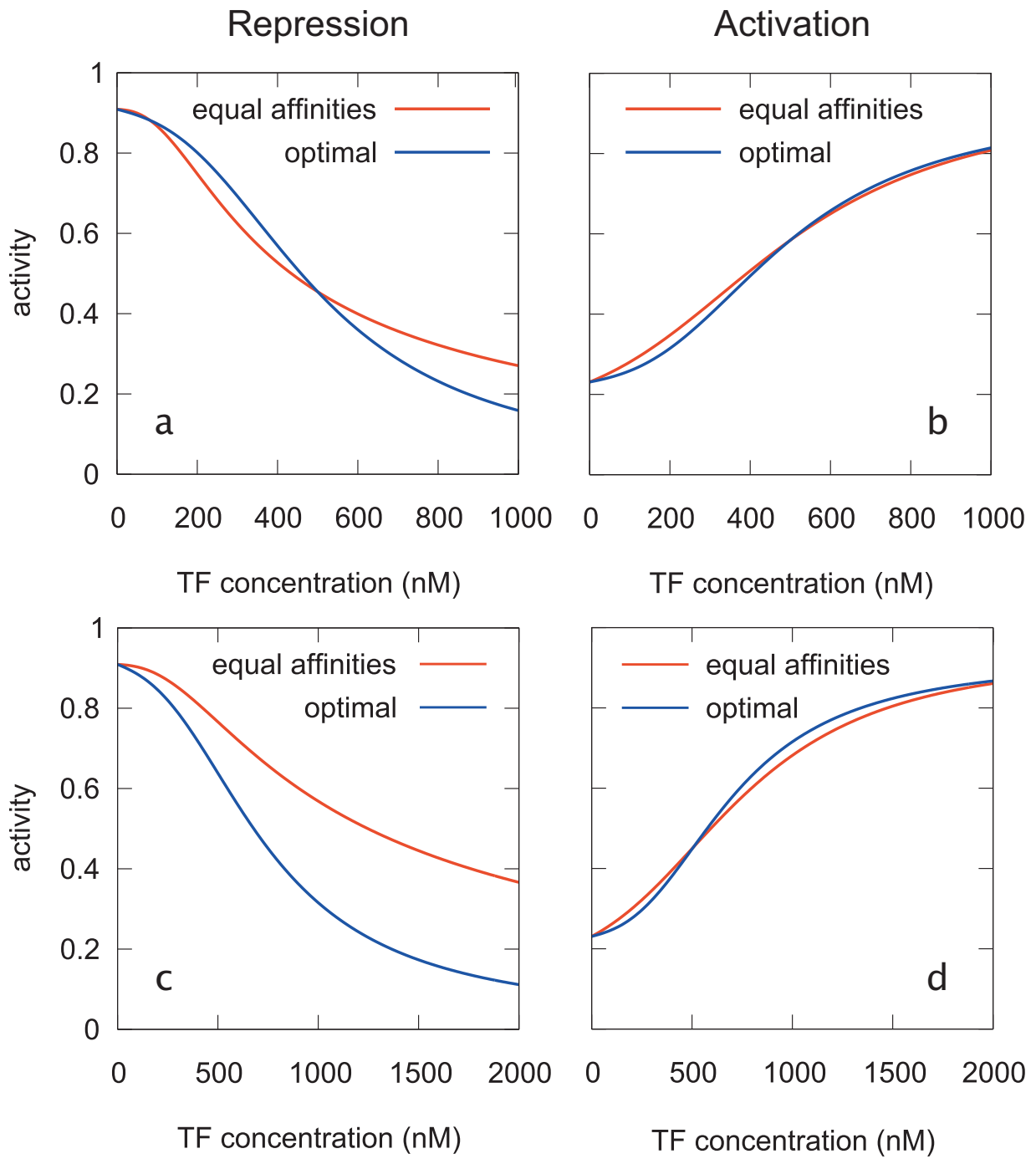


Fig. 2: Response plots of the cooperative repressor (a and c) and activator (b and d) systems. All plots show the responses at $r_2 = r_3 = 1$ (red curves) and the ones for optimized parameters. In figures (a) and (b) we optimized the slope at half maximal repression (a) or half maximal activation (b); we fixed this point at 500 nM. In figure (c) and (d) we optimized the slope at the inflection point of the curve, fixing this point at 500 nM. In the repressor system we chose $q_p = 10$, while in the activation system $q_p = 0.3$; in both cases $\omega = \omega' = 50$. Clearly, steepness of response of the repressor system increases considerably if the relative affinities of the binding sites are fine-tuned. The same holds for the activation system, albeit to a much lesser extent.

### B. Activation

Here we present the case of cooperative activation by two auxiliary TF binding sites. We use the same conventions as in the previous subsection. For this system, the partition sums become:

$$\begin{aligned} z_{\rm on} &= q_p \Bigg(1 + (\omega' + r_2 + r_3)\left(\frac{c_A}{K}\right) \\ &\quad + (r_2 r_3 \omega + r_2 \omega\omega' + r_3\omega')\left(\frac{c_A}{K}\right)^2 \\ &\quad + r_2 r_3 \omega^2 \omega' \left(\frac{c_A}{K}\right)^3\Bigg) \\ z_{\rm off} &= 1 + (1 + r_2 + r_3)\left(\frac{c_A}{K}\right) \\ &\quad + (r_2\omega + r_3 + r_2 r_3 \omega)\left(\frac{c_A}{K}\right)^2 \\ &\quad + r_2 r_3 \omega^2 \left(\frac{c_A}{K}\right)^3, \end{aligned}$$

where $c_A$ is the concentration of the activator TF.

Again we use three different measures for the steepness of response.

1. We optimize the slope $s_{\rm half}$ at the concentration $c_{\rm half}$ at with the expression level is half maximal. We adjust $K$ such that $c_{\rm half} = 500$ nM. The results are shown in Fig. 2b, where we use $\omega = \omega' = 50$ and $q_p = 0.3$. The optimal parameter set, $r_2 = 1.74$ and $r_3 = 12.3$ provides an increase in $s_{\rm half}$ of 11%. Note that the affinity of $O_3$ is much higher than those of the other sites.

2. We fit the plots to the Hill function defined as:

$$H_A(c_A) = A \frac{f^{-1} + \left(\frac{c_A}{K}\right)^n}{1 + \left(\frac{c_A}{K}\right)^n} \qquad (10)$$

   The results (not shown) are very similar to those obtained by the previous method. The gain in terms of $n$ is a modest 11%.

3. We maximize the slope $s_{\rm inf}$ at the inflection points of the plots, adjusting $K$ such that $c_{\rm inf} = 500$ nM (Fig. 2d). Optimally, $r_2 = 1.97$ and $r_3 = 14.4$, which results in a 27% increase in $s_{\rm inf}$.

The results show that, in order to be optimal, the auxiliary activation sites need to be as strong or stronger than the primary site. This is in stark contrast with the results for homo-cooperative repression, where we saw that the auxiliary sites need to be weak. Also, the site furthest removed from the core promoter has the highest affinity, as we found in our simulations. The increase in steepness resulting from the tuning of the binding affinities, however, is rather modest. Whether in real genetic systems the selection pressure for steep activation is usually strong enough to attain and maintain the optimal affinity ratios in a selection-mutation balance, is unclear.

## IV. MINIMAL MODELS FOR THE COMPLEX GATES

Some of the gates that resulted from the simulations have a rather complex design. Here, we describe simplified quantitative models for the EQU gate and the XOR gate; a simplified description provides more insight into their essential features. The other gates can be described in a similar manner.

### A. The EQU gate

For the EQU gate, the essential ingredients of our minimal model are: a strong promoter, homo-cooperative repression for each of the two TFs, and hetero-cooperative activation when both TFs are present. For simplicity, we make the following assumptions:

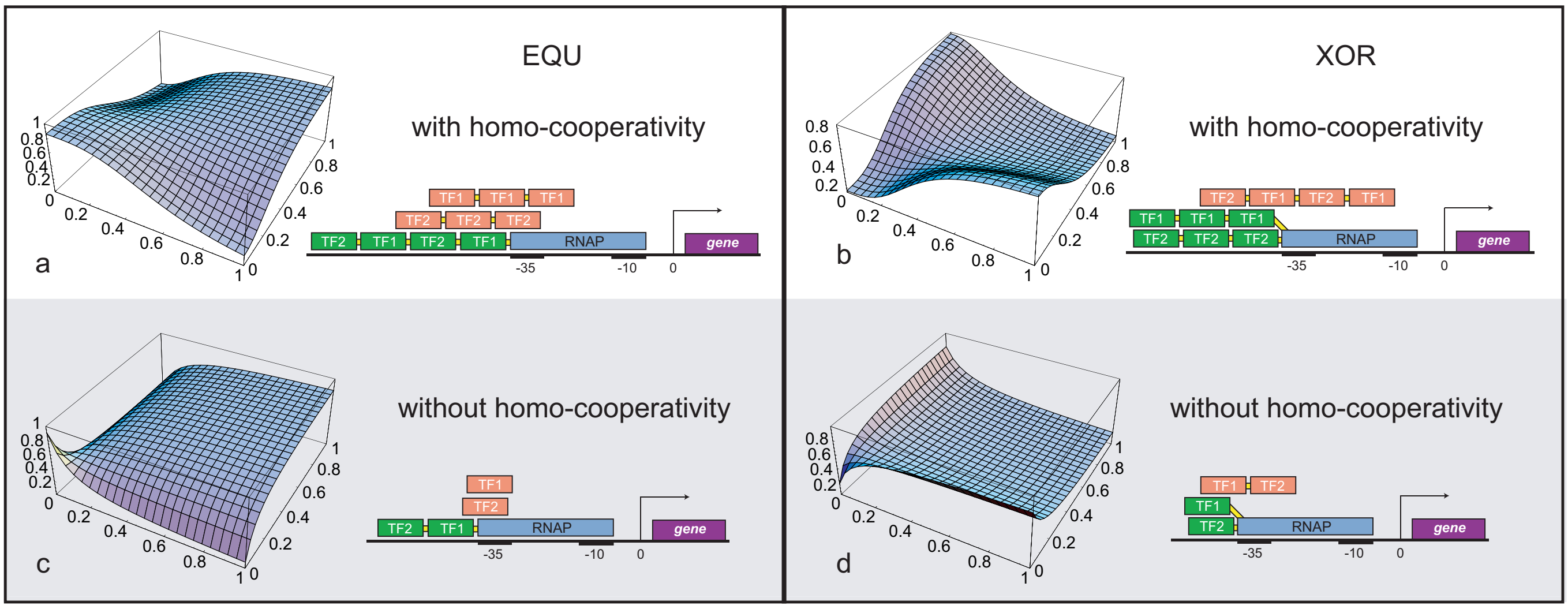


Fig. 3: Response plots resulting from the simplified models of XOR and EQU gates. The concentration units are $\mu$M. (a) EQU gate with the following parameters: $q_p = 6$, $K_a = 11\mu$M, $K_r = 3\mu$M, $n_{a,1} = n_{a,2} = 2$, $n_r = 3$ and $\omega = 30$. (b) An EQU gate without homo-cooperative repression modules ($n_{a,1} = n_{a,2} = n_r = 1$). Note that, although the values in the corners of the plot are consistent with an EQU gate, the full performance is poor. This shows that the complex behavior of the EQU gate requires homo-cooperative modules. Further parameters are: $q_p = 10$, $K_a = 3\mu$M, $K_r = 0.01\mu$M and $\omega = 30$. (c) XOR gate with parameters $q_p = 0.2$, $K_a = 7\mu$M, $K_r = 4\mu$M, $n_{r,1} = n_{r,2} = 2$, $n_a = 3$ and $\omega = 30$. (d) Typical XOR gate with no homo-cooperative activation ($n_{r,1} = n_{r,2} = n_a = 1$). The gate could hardly be classified as an XOR gate, showing that homo-cooperative activation is essential to obtain reasonable XOR gates.

1. All repression sites have an equal dissociation constant $K_r$; all activation sites have dissociation constant $K_a$.
2. The number of sites in each homo-cooperative repression module is the same and equal to $n_r$; the number of sites for the TF $\alpha$ in the hetero-cooperative activation module is $n_{a,\alpha}$.
3. We neglect states in which incomplete modules are bound; of a module either all sites or none of the sites are occupied.
4. The modules exclude each other on the DNA: only one of the modules can be bound at a time.
5. We assume that the TFs bind to their specific binding sites only; we thus neglect the affinities for the other binding sites on the DNA.

For this minimal model we can compute the partition sums as follows:

$$\begin{aligned} Z_{\rm off} &= 1 + (q_{r,1})^{n_r}\omega^{n_r-1} + (q_{r,2})^{n_r}\omega^{n_r-1} \\ &\quad + (q_{a,1})^{n_{a,1}}(q_{a,2})^{n_{a,2}}\omega^{n_{a,1}+n_{a,2}-1}, \qquad (11) \\ Z_{\rm on} &= q_p\left(1 + \omega'(q_{a,1})^{n_{a,1}}(q_{a,2})^{n_{a,2}}\omega^{n_{a,1}+n_{a,2}-1}\right) \qquad (12) \end{aligned}$$

Here we used:

$$q_{r,\alpha} = \frac{c_\alpha}{K_r}, \qquad (13)$$

$$q_{a,\alpha} = \frac{c_\alpha}{K_a}. \qquad (14)$$

Note that in $Z_{\rm off}$ we not only count states in which the repression modules are bound, but also states in which the activation sites are occupied by TFs (but with no RNAP bound). Note also that $Z_{\rm off}$ and $Z_{\rm on}$ are bivariate polynomials in the concentrations $c_\alpha$. The order of these polynomials is determined by the number of binding sites in the modules; the coefficients of each term are set by the dissociation constants. Equation 9 shows that $p_{\rm on}$ can be written in terms of the ratio of these polynomials.

We now consider the design constraints for obtaining an input-output relation that corresponds to an EQU gate. To this end, we first consider the limit in which one of the TFs is present in much larger concentration than the other. An EQU gate requires that in this limit, the expression level, and thus $p_{\rm on}$, should be low. When the concentration $c_1$ is kept constant and $c_2$ is increased, $p_{\rm on}$ approaches a limit value that is determined by the terms of highest order in $c_2$ in $Z_{\rm off}$ and $Z_{\rm on}$. It is given by

$$\lim_{c_2\to\infty} p_{\rm on} = \begin{cases} \frac{\omega' q_p}{1+\omega' q_p} & \text{if } n_r < n_{a,2}, \\ \frac{\omega' q_p (c_1)^{n_{a,1}}}{(\frac{k_a}{k_r})^{n_r}(\frac{k_a}{\omega})^{n_{a,1}} + (c_1)^{n_{a,1}}(1+\omega' q_p)} & \text{if } n_r = n_{a,2}, \\ 0 & \text{if } n_r > n_{a,2}. \end{cases}$$

If $n_r < n_{a,2}$, then $p_{\rm on}$ will approach unity, instead of zero as required: since for the EQU gate the promoter should be strong, $\frac{\omega' q_p}{1+\omega q_p} \approx 1$. If $n_r = n_{a,2}$, the expression level depends on $K_a$, $K_r$, $n_r$ and $n_{a,1}$ ; a judicious choice of their value can allow for an expression level that is consistent with an EQU gate. If, however, $n_r > n_{a,2}$, then

the expression level in the above limit is much more robust to the precise parameter values: if the concentration of TF1 is kept constant, then at sufficiently high concentration of TF2, TF2 will always repress transcription, as required for an EQU gate.

We now consider the scenario in which both concentrations become large. If we keep $c_1 = c_2$ and increase both concentrations, then, as long as $(n_{a,1} + n_{a,2}) > n_r$, the limit value is again

$$\lim_{c_1,c_2\to\infty} p_{\rm on} = \frac{\omega' q_p}{1+\omega' q_p}. \quad (15)$$

A good way to construct an EQU gate is therefore to choose the modules such that $(n_{a,1} + n_{a,2}) > n_r$ (so that the operon is transcribed when $c_1$ and $c_2$ are both high), but to take $n_r > n_{a,1}$ and $n_r > n_{a,2}$ (so that the operon is repressed when only one of the two TF concentrations is high). One obvious choice is $n_{a,1} = n_{a,2} = 2$ and $n_r = 3$. This result is shown in Fig. 3a. It is seen that this gate can indeed be classified as an EQU gate.

The EQU gate that results from our simulations (see Fig. 4 in the main text) deviates slightly from this design (Fig. 3a): the number of repressor sites of TF1 is higher than expected on the basis of the assumptions of the minimal model, so that the requirement $(n_{a,1} + n_{a,2}) > n_r$ is not fulfilled. However, there are three points worthy of note: 1) most of the repressor sites are very weak; the extra repressor sites only play a major role at much higher TF concentrations than shown in Fig. 5; 2) the assumption of the minimal model (Eqs. 11 and 12) that the modules mutually exclude each other completely, while instructive, is not entirely consistent with the assumptions underlying the full model discussed in the main text: it is possible for the complete hetero-cooperative activation module to bind, while simultaneously the repressor sites that do not overlap with the activation module, are also occupied; 3) while the previous points concern the simplicity of the assumptions of the minimal model, this point is more fundamental. In our simulations, we selected the gates not just based on their behavior in the limits of high concentrations: we also selected for a *steep* repression curve. The resulting gate is thus a compromise between the requirement of a steep response – favoring a high number of repression sites – and maximal activation when both TFs are present.

Fig. 3c shows the result for an EQU gate with no homo-cooperative repression modules ($n_{a,1} = n_{a,2} = n_r = 1$). In the limit that $c_1 \to 0$ and $c_2 \to \infty$ and in the limit that $c_1 \to \infty$ and $c_2 \to 0$, the expression level approaches zero, as required for an EQU gate. Nevertheless, the input-output relation differs markedly from the gate with homo-cooperativity (Fig. 3a); indeed, one could argue that the gate without homo-cooperativity does not classify as an EQU gate. This shows that homo-cooperativity does not only allow for a steep response, but also can play an important role in signal integration.

### B. The XOR gate

For the XOR gate, the essential ingredients of the minimal model are: a weak promoter, homo-cooperative activation by each of the two TFs, and hetero-cooperative repression when both TFs are present. We make the same simplifying assumptions as in the previous section. However, here the number of sites in each of the activation complexes is denoted by $n_a$, while the number of sites of TF $\alpha$ in the hetero-cooperative repression complex is $n_{r,\alpha}$. This results in the following expressions:

$$\begin{aligned} Z_{\rm off} &= 1 + (q_{r,1})^{n_{r,1}} (q_{r,2})^{n_{r,2}} \omega^{n_{r,1}+n_{r,2}-1} \\ &\quad + (q_{a,1})^{n_a} \omega^{n_a-1} + (q_{a,2})^{n_a} \omega^{n_a-1}, \quad (16) \\ Z_{\rm on} &= q_p \left(1 + \omega' (q_{a,1})^{n_a} \omega^{n_a-1} + \omega' (q_{a,2})^{n_a} \omega^{n_a-1}\right) \quad (17) \end{aligned}$$

When both TFs are absent, the operon should be off; therefore a XOR gate needs a weak promoter. When increasing $c_2$ at constant $c_1$, or $c_1$ at constant $c_2$, activation should occur. The limit value of $p_{\rm on}$ for $c_\alpha \to \infty$ depends on $n_a$ and $n_{r,\alpha}$; if $n_a > n_{r,\alpha}$, activation wins the competition with repression. In the limit of high concentrations of both TFs ($c_1 = c_2$, $c_1 \to \infty$), the XOR should be off. This is satisfied if $(n_{r,1} + n_{r,2}) > n_a$. One option is therefore to choose $n_{r,1} = n_{r,2} = 2$ and $n_a = 3$. Fig. 3 shows the result for this minimal model.

The XOR gate that results from our simulations (see Fig. 4 and 5 of the main text), again deviates slightly from this design. The number of repressor sites of TF2 is higher than anticipated, so that the requirement $n_a > n_{r,2}$ is not fulfilled. As for the EQU gate, on the one hand this is due to the simplicity of the minimal model, while on the other hand it is a result of the selection for a steep response.

Fig. 3d shows the result for an XOR gate without homo-cooperative activation modules – the activation when either TF1 or TF2 is present, is non-cooperative ($n_{r,1} = n_{r,2} = n_a = 1$). It is seen that the performance of the gate is poor. This again shows that homo-cooperativity can be a useful mechanism for shaping complex input-output relations.

[1] Mandel-Gutfreund Y, Margalit H (1998) Quantitative parameters for amino acid-base interactions: implications for prediction of protein-DNA binding sites. NAR 26(10):2306–2312.

[2] Lisser S, Margalit H (1993) Compilation of *E.coli* mRNA promoter sequences. NAR 21(7):1507–1516.

[3] Berg OG, von Hippel PH (1987) Selection of DNA binding sites by regulatory proteins. statistical-mechanical theory and application to operators and promoters. J Mol Biol 193:723–750.

[4] Berg OG (1988) Selection of DNA binding sites by regulatory proteins. functional specificity and pseudosite competition. J Biomol Struc & Dynam 6(2):275–297.

[5] Berg OG, von Hippel PH (1987) Selection of DNA binding sites by regulatory proteins ii. the binding specificity of Cyclic AMP receptor protein to recognition sites. J Mol Biol 193:723–750.

[6] Buchler NE, Gerland U, Hwa T (2003) On schemes of combinatorial transcription logic. Proc Natl Acad Sci USA 100(9):5136–5141.

[7] Shea MA, Ackers GK (1985) The $O_R$ control system of bacteriophage lambda. a physical-chemical model for gene regulation. J Mol Biol 181:211–230.

[8] Bintu L, Buchler NE, Garcia HG, Gerland U, Hwa T, et al. (2005) Transcription regulation by the numbers 1: Models. Curr Opin Gen & Dev 15:116–124.

[9] Bintu L, Buchler NE, Garcia HG, Gerland U, Hwa T, et al. (2005) Transcription regulation by the numbers 2: Applications. Curr Opin Gen & Dev 15:125–135.

[10] Graham I, Duke T (2005) The logical repertoire of ligand-binding proteins. Phys Biol 2:159–165.
